# Relationship between occupational injuries and the provision of safety and health information: data from the 4th Korean working conditions survey

**DOI:** 10.1186/s40557-018-0247-7

**Published:** 2018-06-04

**Authors:** Ju-il Seo, Gab-Sik Shin, Min Gi Kim, Young-Sun Min

**Affiliations:** Department of Occupational and Environmental Medicine, Dongguk University Gyeongju Hospital, Gyeongju-si, Gyeongsangbuk-do Republic of Korea

**Keywords:** Safety and health information, Occupational injury, KWCS

## Abstract

**Background:**

The aim of this study was to examine the relationship between the provision of safety and health information (PSHI) and occupational injuries.

**Methods:**

This study was based on data from the 4th Korean Working Conditions Survey (KWCS) (2014). The sample consisted of data from 24,527 wage workers and was divided into high-risk and low-risk groups, depending on the probability of occupational injury. The high-risk group included subjects who could cause harm to themselves or others due to errors during work. We applied chi-squared tests and logistic regression analyses to examine the relationship between PSHI and occupational injuries.

**Results:**

In the high-risk group, workers with no PSHI showed an adjusted odds ratio of 1.81 for occupational injury (95% CI 1.33–2.47). In contrast, there was no statistically significant relationship between PSHI and the incidence of occupational injury in the low-risk group.

**Conclusions:**

To prevent occupational injuries, multi-faceted approaches that take different levels of injury risk into account are needed. Among workers with a high risk of occupational injury, more a stringent safety education program is required.

## Background

Occupational injuries do not only have serious personal effects, but they can also result in loss of life and/or property. In the case of industrial accidents in South Korea, direct and indirect economic losses in 2015 were estimated at 20.3 trillion Korean won [1]. This was an increase of 3.89% from 19.6 trillion won in losses in 2014, indicating an increasing trend despite a decrease in the industrial accident rate [1]. In order to reduce such economic damage related to occupational injuries, the Korea Occupational Safety & Health Agency has been operating an “Occupational Acute Intoxication and Injury Management System” in Incheon City [2]. That system was designed to report suspected cases of occupationally acute poisoning, as well as to share case information, conduct field surveys, and undertake epidemiologic surveys at regional intervention centers. In addition to such follow-up efforts, the prevention of injuries and diseases is also important. Many researchers and policy-makers have recognized that occupational and non-occupational factors may simultaneously contribute to worker safety and health [3, 4]. A primary step in the prevention of disease not only includes improvement of workers’ health but also identification and elimination of various risk factors for occupational diseases [5]. Several types of health information and data from the behavioral sciences have contributed to reducing mortality and morbidity as well as injury- or disease-related complications [6]. Therefore, prevention of occupational injuries and diseases, as well as the provision of safety and health information (PSHI), are essential for all workers. The Korean Occupational Safety and Health (OSH) Act was enacted in 1981, and since a revision in 1990, there has been promotion of a systematic workplace health management project. A number of papers related to occupational health education practices and occupational health care providers have been published, but most have focused on manufacturing or secondary industries [7]. An interest in occupational health has spread to various fields, but the importance of and concern about PSHI have not been sustained [7]. For example, in 2004, only 56.1% of workers received safety and health education as mandatorily required by Korean OSH Act [8].

The aim of this study was to clarify the relationship between PSHI and occupational injuries in a nationally representative sample of South Korean workers. Prior research in Korea has rarely focused on the association between the rate of occupational injuries and PSHI, in particular, with respect to the degree of occupational-injury risks. In this study, we examined various influencing elements, including general characteristics, occupational characteristics, and job-related factors, that are associated with occupational injuries.

## Methods

### Study subjects

This study analyzed data from the 4th Korea Working Conditions Survey (KWCS) (2014) [9]. The Occupational Safety and Health Research Institute (OSHRI) has been conducting the KWCS since 2006 in South Korea. The KWCS emulated the European Working Conditions Survey and the UK Labor Force Survey to identify the overall South Korean work conditions such as employment type, job stability, occupation, and risk factor exposure. Among households from the 2010 Population and Housing Census [10], the KWCS selected individuals who met the criteria of being an “employee,” who were laborers, 15 years or older, and who worked for more than 1 h per week at the time of the survey. Trained interviewers visited the subjects’ homes and conducted one-to-one interviews. Statistics Korea determined the KWCS information’s reliability to increase the usage of its data. The survey’s response rate was 33.0%, the cooperation rate 69.9%, and the refusal rate 14.2% [11]. Because the characteristics of self-employed and wage workers are markedly different, this study restricted the subjects to wage workers. Out of a total of 50,007 respondents, 30,751 were paid workers, excluding military persons. A final sample of 24,527 persons was selected after excluding 6224 persons with missing data or refusals in responding to items necessary for the analyses. We compared the control group with the subjects with missing values, and there was no statistically significant difference in their PSHI and occupational-injury characteristics (*p* = 0.791 and 0.357, respectively).

### General characteristics

The subjects’ general characteristics included sex, age, education level, and monthly household income. The educational level was divided into three groups: (1) middle school or below, (2) high school, and (3) college or above. The monthly household income level was categorized as follows: less than 1.5 million Korean won (KRW), 1.5–2.49 million KRW, 2.5–3.99 million KRW, and 4 million KRW and above.

### Occupational characteristics

In the KWCS, occupational types were divided into 11 groups according to the 6th Korean Standard Classification of Occupations. In this study, those groups were re-classified into three groups: (1) white-collar (manager, professional, technicians and associate professionals, office workers), (2) pink-collar (service workers, sales workers), and (3) blue-collar (skilled agricultural and fishery workers, craft workers and those of related trades, plant and machine operators and assemblers, elementary occupations). Company size was included as an occupational characteristic and was defined by the number of employees. The occupational characteristics also included working hours per week, tenure, shift work status, type of employment, and the presence of labor unions. Working hours were classified according to the South Korean Labor Standards Act, with 52 h as the standard, comprised of 40 working hours and 12 overtime working hours.

### Job-related factors

Risk factors were classified into three categories: (1) physical risk factors (vibrations, noise, high and low temperatures), (2) biochemical risk factors (breathing in smoke or fumes, breathing in vapors such as solvents and thinners, exposure to chemical agents or infectious materials), and (3) ergonomic risk factors (tiring or painful positions, lifting or moving people, carrying or moving heavy loads, standing, repetitive hand or arm movements). Being exposed to risk factors was defined as exposure for approximately ≥1/4 of the work hours. Usage of personal protective equipment (PPE) was categorized as those who did not require it (no need), those who required it and always wore it (need/wear), and those who required it but did not wear it (need/no wear).

### Provision of safety and health information (PSHI)

A positive PSHI status was present if the subject responded “Very well informed” or “Well informed” to the question “Regarding the health and safety risks related to performance of your job, how well informed would you say you are?”

### Occupational injuries and injury risk assessment

Occupational injuries were assessed by using the question “Over the last 12 months, did you suffer from any of the following health problems?” and the sub-question “Injuries (be hurt by accident).” Anyone who answered “Yes” was considered as having had one or more injuries. Those who responded with “Yes” to the question “If there was an injury, was it related with your job?” were defined as having experienced occupational injuries. We also assessed the injury risk at the workplace. Belonging to the high-risk group for occupational injuries was determined if the answer was “Always,” “Most of the time,” or “Sometimes” to the question “If you make mistakes in your work, could it cause…” with the sub-question “Physical injury to yourself” or “Physical injury to other people.” If the answer was “Rarely” or “Never,” the subject was classified as a low-risk group member for occupational injuries.

### Statistical analyses

Chi-squared tests were used to determine the relevance of the subjects’ characteristics to the PSHI status and occupational injuries. General characteristics, occupational characteristics, job-related factors, and the PSHI were independent variables. The main dependent variables included occupational injuries and the PSHI; the latter used to examine poorly informed groups. Crude (unadjusted) and adjusted odds ratios (ORs) were calculated via multivariate logistic regression analyses to estimate the association between PSHI and occupational injuries. The ORs were adjusted for variables which showed a statistically significant association with occupational injury among general characteristics (age, sex, education level, monthly household income) (Model I), or occupational characteristics (working hours per week, tenure, existence of labor unions) and job-related factors (physical risk factors, biochemical risk factors, PPE) (Model II). Study subjects were divided into high-risk and low-risk groups, depending on the probability of occupational injuries. All analyses were performed by using SPSS ver. 20.0 (SPSS Inc., Chicago, IL, USA) after stratifying the data by the risk of occupational injuries. The statistical significance was set at *p* < 0.05. We used the original data to show the number (N) of people and applied weighted analyses to display the overall proportions (%) as well as the *p*-values.

## Results

### 1. Characteristics of study subjects depending on the risk of occupational injuries

Of the 24,527 subjects, 74.1% were placed in the low-risk group and 25.9% were placed in the high-risk group. Statistically significant differences between the two groups were observed for age, sex, education level, monthly income, working hours per week, tenure, shift work, type of employment, occupational type, risk factor exposure, and PPE use, with the significance of the differences in the last three variables being particularly pronounced. There were no statistically significant differences between the two groups in the number of employees or the presence of labor unions. The overall occupational-injury rate was 1.0%, and the injury rate differed significantly between the low-risk (0.4%) and high-risk (3.1%) groups (Table 1).

**Table 1:** Characteristics of study subjects according to occupational injury risk

Variables	Total		Risk of occupational injury				
			Low-risk		High-risk		*p*-value^c^
	N^a^	%^b^	N^a^	%^b^	N^a^	%^b^	
Total	24,527	100	18,177	74.1	6350	25.9	
General characteristics							
Age (years)							
15–29	3454	13.6	2746	14.6	708	10.7	< 0.001
30–39	6151	26.3	4811	27.9	1340	21.3	
40–49	7092	29.1	5284	29.2	1808	28.8	
50–59	4920	19.7	3349	17.9	1571	25.1	
≥ 60	2910	11.3	1987	10.4	923	14.1	
Sex							
Male	12,639	51.8	8640	47.8	3999	64.1	< 0.001
Female	11,888	48.2	9537	52.2	2351	35.9	
Education level							
Middle school or below	2996	11.3	1887	9.4	1109	17.0	< 0.001
High school	9455	36.8	6266	32.4	3189	50.0	
College or above	12,076	52.0	10,024	58.2	2052	33.1	
Monthly income (KRW 10,000)							
< 150	7435	27.3	5501	27.1	1934	28.0	< 0.001
150–249	8510	35.0	6198	34.2	2312	37.3	
250–399	6467	28.3	4773	28.4	1694	27.9	
≥ 400	2115	9.4	1705	10.3	410	6.7	
Occupational characteristics							
Occupational type							
White-collar	10,228	44.9	8943	52.5	1285	21.8	< 0.001
Pink-collar	6441	24.2	4865	24.8	1576	22.5	
Blue-collar	7858	30.9	4369	22.8	3489	55.7	
Number of employees							
1–4	5495	20.5	4047	20.3	1448	21.3	0.186
5–49	12,514	52.2	9302	52.3	3212	51.8	
50–299	4459	19.1	3317	19.3	1142	18.5	
≥ 300	2059	8.2	1511	8.1	548	8.3	
Working hours per a week (hours)							
< 40	13,301	52.9	10,350	55.5	2951	44.9	< 0.001
41–52	6796	29.3	4955	29.2	1841	29.5	
53–60	3087	12.5	2011	10.8	1076	17.6	
≥ 61	1343	5.4	861	4.6	482	7.9	
Tenure (years)							
< 1	3018	11.6	2203	11.3	815	12.5	< 0.001
1–5	8846	37.2	6713	38.1	2133	34.4	
≥ 5	12,663	51.2	9261	50.6	3402	53.1	
Shift work							
Yes	2551	10.0	1643	8.6	908	14.5	< 0.001
No	21,976	90.0	16,534	91.4	5442	85.5	
Type of employment							
Regular	18,188	76.1	13,841	78.1	4347	69.8	< 0.001
Temporary	6339	23.9	4336	21.9	2003	30.2	
Labor unions							
Presence	3441	14.3	2506	14.2	935	14.8	0.219
Absence	21,086	85.7	15,671	85.8	5415	85.2	
Job-related factors							
Physical risk factors							
Yes	9467	37.4	5243	27.9	4224	66.6	< 0.001
No	15,060	62.6	12,934	72.1	2126	33.4	
Biochemical risk factors							
Yes	6097	24.0	3017	15.8	3080	49.0	< 0.001
No	18,430	76.0	15,160	84.2	3270	51.0	
Ergonomic risk factors							
Yes	20,495	81.8	14,438	77.5	6057	94.9	< 0.001
No	4032	18.2	3739	22.5	293	5.1	
Usage of personal protective equipment							
No need	18,510	76.7	15,468	86.2	3042	47.8	< 0.001
Need/wear	5478	21.2	2440	12.4	3038	47.8	
Need/no wear	539	2.2	269	1.4	270	4.4	
Provision of safety and health information							
Yes	15,623	64.2	11,227	62.3	4396	70.1	< 0.001
No	8904	35.8	6950	37.7	1954	29.9	
Occupational injuries							
Yes	276	1.0	82	0.4	194	3.1	< 0.001
No	24,251	99.0	18,095	99.6	6156	96.9	

^a^Unweighted case numbers of workers

^b^Percentages based on weighted analysis

^c^Derived by weighted chi-squared test

### 2. Provision of safety and health information (PSHI)

The overall proportion of the study population with PSHI was 64.2%, and there was a significant difference in PSHI status between the low-risk group (62.3%) and the high-risk group (70.1%) (Table 1). There were significant differences between all subjects’ characteristics and PSHI status, which depended on the risk of occupational injury, but, with regard to age, there was no significant relationship in either risk group. Women had a statistically significant lower PSHI status than men in both groups. The lower the monthly income, the lower the PSHI status, and the smaller the company (i.e., fewer employees), the lower the PSHI status (*p* for trend < 0.001 for both associations). With regard to occupational types, PSHI status was lowest in the pink-collar group, a group that includes sales workers and service workers. PSHI status was significantly lower in the group without unions (61.7%) than in the group with unions (79.7%). Among the job-related factors, the group with the need to wear PPE had a higher PSHI status than the group that did not need to wear it. In addition, the PSHI was significantly higher in the PPE-wearing group than in those who did not wear a PPE (Table 2).

**Table 2:** Relationships between characteristics of study subjects and PSHI by injury risk level

Variables	PSHI (Low-risk group)				PSHI (High-risk group)			
	Total	Yes		*p*-value^c^	Total	Yes		*p*-value^c^
	N^a^	N^a^	%^b^		N^a^	N^a^	%^b^	
General characteristics								
Age (years)								
15–29	2746	1541	57.6	< 0.001	708	459	67.5	< 0.001
30–39	4811	3053	63.7	0.168^d^	1340	973	72.8	0.039^d^
40–49	5284	3369	63.9		1808	1280	71.6	
50–59	3349	2087	62.3		1571	1084	69.8	
≥ 60	1987	1177	60.2		923	600	65.6	
Sex								
Male	8640	5757	66.2	< 0.001	3999	2976	74.4	< 0.001
Female	9537	5470	58.7		2351	1420	62.5	
Education level								
Middle school or below	1887	1064	58.5	< 0.001	1109	708	65.3	< 0.001
High school	6266	3512	56.7	< 0.001^d^	3189	2179	69.1	< 0.001^d^
College or above	10,024	6651	66.0		2052	1509	74.1	
Monthly income (KRW 10,000)								
< 150	5501	2872	53.8	< 0.001	1934	1148	61.1	< 0.001
150–249	6198	3816	61.6	< 0.001^d^	2312	1555	67.8	< 0.001^d^
250–399	4773	3261	67.2		1694	1345	78.9	
≥ 400	1705	1278	73.4		410	348	84.2	
Occupational characteristics								
Occupational type								
White-collar	8943	6050	67.4	< 0.001	1285	1024	79.8	< 0.001
Pink-collar	4865	2465	51.6		1576	905	59.9	
Blue-collar	4369	2712	61.9		3489	2467	70.5	
Number of employees								
1–4	4047	1887	48.5	< 0.001	1448	776	56.3	< 0.001
5–49	9302	5723	61.8	< 0.001^d^	3212	2181	68.5	< 0.001^d^
50–299	3317	2398	71.3		1142	935	80.9	
≥ 300	1511	1219	77.9		548	504	91.4	
Working hours per a week (hours)								
< 40	10,350	6479	63.0	< 0.001	2951	2072	71.7	< 0.001
41–52	4955	3117	63.1	< 0.001^d^	1841	1326	72.1	< 0.001^d^
53–60	2011	1152	58.2		1076	697	66.4	
≥ 61	861	479	57.1		482	301	61.9	
Tenure (years)								
< 1	2203	1132	52.2	< 0.001	815	518	67.2	< 0.001
1–5	6713	3956	59.5	< 0.001^d^	2133	1379	65.0	< 0.001^d^
≥ 5	9261	6139	66.6		3402	2499	74.1	
Shift work								
Yes	1643	1117	67.0	< 0.001	908	745	80.9	< 0.001
No	16,534	10,110	61.8		5442	3651	68.3	
Type of employment								
Regular	13,841	8997	64.6	< 0.001	4347	3185	73.3	< 0.001
Temporary	4336	2230	53.8		2003	1211	62.7	
Labor unions								
Presence	2506	1978	77.4	< 0.001	935	813	86.1	< 0.001
Absence	15,671	9249	59.8		5415	3583	67.3	
Job-related factors								
Physical risk factors								
Yes	5243	3452	65.8	< 0.001	4224	3035	72.3	< 0.001
No	12,934	7775	60.9		2126	1361	65.8	
Biochemical risk factors								
Yes	3017	2012	65.7	< 0.001	3080	2255	73.2	< 0.001
No	15,160	9215	61.6		3270	2141	67.1	
Ergonomic risk factors								
Yes	14,438	8794	61.5	< 0.001	6057	4171	69.7	0.004
No	3739	2433	65.1		293	225	77.1	
Usage of personal protective equipment								
No need	15,468	8866	58.3	< 0.001	3042	1612	54.4	< 0.001
Need/wear	2440	2179	89.4		3038	2621	86.5	
Need/no wear	269	182	66.3		270	163	62.1	

^a^Unweighted case numbers of workers

^b^Percentages based on weighted analysis

^c^*P*-value of the weighted chi-squared test results

^d^*P*-value for trend in the weighted analysis

### 3. Occupational Injuries

There were various differences between the low-risk group and the high-risk group with regard to occupational injuries. The characteristics that showed statistically significant relationships with occupational injuries, regardless of risk group, were age, monthly income, working hours per week, physical and chemical risk factors, as well as PPE status. In contrast, there were no statistically significant relationships between occupational injuries and sex, the number of employees, or the shift work status in either group. In the low-risk group, the higher the age and the lower the education level, the higher was the number of occupational injuries (*p* for trend< 0.001), while there were no significant relationships in the high-risk group. Among the occupational types, the low-risk group had the highest number of occupational injuries in the blue-collar category, whereas it was highest in pink-collar workers in the high-risk group. Among all study subjects, the occupational injury incidence tended to increase with longer working hours per week. With respect to employment types, there were significantly more occupational injuries in the temporary workers in the low-risk group than for regular workers, but this difference was not statistically significant in the high-risk group. Regardless of injury risk status, workers without labor unions displayed more occupational injuries, although this was only statistically significant in the high-risk group. The number of occupational injuries was high in workers exposed to physical, chemical, and ergonomic risk factors (Table 3).

**Table 3:** Relationship between subject characteristics and occupational injuries by injury risk levels

Variables	Occupational injuries (Low-risk group)				Occupational injuries (High-risk group)			
	Total	Yes		*p*-value^c^	Total	Yes		*p*-value^c^
	N^a^	N^a^	%^b^		N^a^	N^a^	%^b^	
General characteristics								
Age (years)								
15–29	2746	6	0.2	< 0.001	708	11	1.7	0.012
30–39	4811	16	0.3	< 0.001^d^	1340	45	3.2	0.986^d^
40–49	5284	19	0.3		1808	72	4.3	
50–59	3349	21	0.5		1571	44	2.5	
≥ 60	1987	20	0.8		923	22	2.7	
Sex								
Male	8640	38	0.4	0.829	3999	127	3.3	0.466
Female	9537	44	0.4		2351	67	2.7	
Education level								
Middle school or below	1887	24	1.1	< 0.001	1109	31	2.7	0.130
High school	6266	31	0.4	< 0.001^d^	3189	111	3.6	0.393^d^
College or above	10,024	27	0.2		2052	52	2.5	
Monthly income (KRW 10,000)								
< 150	5501	37	0.5	0.018	1934	39	1.9	0.006
150–249	6198	19	0.3	0.053^d^	2312	89	3.9	0.056^d^
250–399	4773	17	0.3		1694	55	3.4	
≥ 400	1705	9	0.3		410	11	2.7	
Occupational characteristics								
Occupational type								
White-collar	8943	25	0.2	< 0.001	1285	28	2.3	0.119
Pink-collar	4865	20	0.3		1576	50	3.5	
Blue-collar	4369	37	0.7		3489	116	3.3	
Number of employees								
1–4	4047	22	0.4	0.599	1448	47	3.1	0.943
5–49	9302	43	0.4	0.216^d^	3212	98	3.3	0.481^d^
50–299	3317	12	0.3		1142	34	3.0	
≥ 300	1511	5	0.2		548	15	2.5	
Working hours per a week (hours)								
< 40	10,350	44	0.3	0.042	2951	54	1.8	< 0.001
41–52	4955	18	0.3	0.021^d^	1841	70	3.7	< 0.001^d^
53–60	2011	11	0.5		1076	53	5.3	
≥ 61	861	9	1.0		482	17	3.5	
Tenure (years)								
< 1	2203	5	0.2	0.168	815	12	1.6	0.019
1–5	6713	36	0.4	0.714^d^	2133	69	3.2	0.012^d^
≥ 5	9261	41	0.4		3402	113	3.4	
Shift work								
Yes	1643	10	0.5	0.318	908	20	2.2	0.107
No	16,534	72	0.4		5442	174	3.3	
Type of employment								
Regular	13,841	48	0.3	< 0.001	4347	132	3.2	0.899
Temporary	4336	34	0.7		2003	62	3.0	
Labor unions								
Presence	2506	8	0.2	0.289	935	19	1.9	0.049
Absence	15,671	74	0.4		5415	175	3.3	
Job-related factors								
Physical risk factors								
Yes	5243	55	0.9	< 0.001	4224	161	3.8	< 0.001
No	12,934	27	0.2		2126	33	1.8	
Biochemical risk factors								
Yes	3017	42	1.2	< 0.001	3080	128	4.0	< 0.001
No	15,160	40	0.2		3270	66	2.2	
Ergonomic risk factors								
Yes	14,438	78	0.5	< 0.001	6057	188	3.1	0.305
No	3739	4	0.1		293	6	2.8	
Usage of personal protective equipment								
No need	15,468	54	0.3	< 0.001	3042	66	1.9	< 0.001
Need/wear	2440	24	0.9		3038	118	4.2	
Need/no wear	269	4	1.6		270	10	4.5	
Provision of safety and health information								
Yes	11,227	47	0.4	0.406	4396	119	2.8	0.016
No	6950	35	0.4		1954	75	3.8	

^a^Unweighted case numbers of workers

^b^Percentages based on weighted analysis

^c^*P*-value of the weighted chi-squared test results

^d^*P*-value for trend in the weighted analysis

### 4. Relationship between PSHI and occupational injuries

There was no statistically significant difference in the low-risk group (*p* = 0.406) regarding the PSHI; however, in the high-risk group, the workers with no PSHI had a high incidence of occupational injuries (*p* = 0.016) (Table 3). After appointing workers with PSHI as the reference group, the ORs of occupational injuries in the high-risk group without PSHI were as follows: Crude (unadjusted) (OR 1.392, 95% CI 1.055–1.837), Model I (adjusted) (OR 1.454, 95% CI 1.095–1.932), and Model II (adjusted) (OR 1.812, 95% CI 1.330–2.468) (Table 4).

**Table 4:** Odds ratios of occupational injuries associated with PSHI in the high-risk group

Variables	Occupational injuries (high-risk group)					
	Crude		Model I^a^		Model II^b^	
	OR	95% CI	OR	95% CI	OR	95% CI
Provision of safety and health information (ref.^c^ Yes) No	1.392	1.055–1.837	1.454	1.095–1.932	1.812	1.330–2.468
Age (years) (ref. 15–29) 30–39			1.560	0.750–3.242	1.420	0.668–3.018
40–49			0.854	0.481–1.517	0.881	0.495–1.569
50–59			0.661	0.392–1.113	0.678	0.402–1.144
≥ 60			1.196	0.711–2.013	1.241	0.736–2.093
Sex (ref. Male) Female			0.953	0.688–1.320	1.337	0.944–1.893
Education level (ref. Middle school or below) High school			0.610	0.354–1.050	0.784	0.455–1.352
College or above			0.649	0.464–0.908	0.802	0.571–1.127
Monthly income (KRW 10,000) (ref. <150) 150–249			1.557	0.758–3.198	1.382	0.662–2.888
250–399			0.715	0.385–1.328	0.853	0.452–1.608
≥ 400			0.808	0.436–1.496	1.013	0.541–1.897
Working hours per a week (hours) (ref. <40) 41–52					1.672	0.982–2.848
53–60					0.934	0.562–1.552
≥ 61					0.708	0.423–1.185
Tenure (years) (ref. <1) 1–5					1.867	1.045–3.338
≥ 5					0.998	0.734–1.356
Labor unions (ref. Yes) No					1.704	1.044–2.781
Physical risk factors (ref. No) Yes					1.435	0.978–2.107
Biochemical risk factors (ref. No) Yes					1.230	0.896–1.688
Usage of personal protective equipment (ref. No need) Need/wear					2.030	1.096–3.760
Need/no wear					0.828	0.463–1.480

^a^Adjusted for general characteristics (age, sex, education level, monthly income)

^b^Adjusted for general characteristics (age, sex, education level, monthly income), occupational characteristics (working hours per a week, tenure, labor unions), and job-related factors (physical risk factors, biochemical risk factors, personal protective equipment)

^c^Reference group

## Discussion

This study was conducted to examine the relationship between PSHI at the workplace and occupational injuries in a large-scale nationally representative sample of South Korean workers. We hypothesized that the importance of PSHI would differ depending on the risk of occupational injuries. Therefore, to determine the significance of PSHI related to occupational injury risk level, and because various characteristics of workers differed depending on their risk of occupational injury, the subjects were divided into two groups based on injury risk. In the high-risk group, there was a statistically significant relationship between PSHI and occupational injuries, and the occupational injury rate of workers without PSHI was up to 1.81 times higher than the rate in those with PSHI. Therefore, the better the safety information provided at the workplace, the fewer work-related injuries occurred, which is consistent with the results in previous studies. For example, a study that analyzed the characteristics of Korean wage workers during the 1st KWCS, conducted in 2006, showed that the odds ratio of occupational injuries in the group without PSHI was 1.29 (95% CI 1.05–1.59) [7]. In another investigation that assessed occupational injuries in Korea with health insurance and industrial accident insurance information, the rate of occupational injuries was higher in those who did not receive accident prevention education at the workplace than in those who did (OR 1.62, 95% CI 1.42–1.84) [12]. In addition, according to Ghosh et al., poor safety performance by workers was significantly associated with occupational injuries (adjusted OR 3.10, 95% CI 1.45–6.63) [13]. Moreover, in an overview which summarized ten studies on the association between organizational and workplace factors with injury rates, an active role of top management in health and safety was associated with lower injury rates [14]. Thus, safety education and training within the workplace function as precautionary measures against occupational diseases, such as hearing and vision loss, as well as against occupational injuries [15, 16].

Statistically significant relationships between workers’ characteristics and PSHI status were detected in almost all characteristics analyzed in this study. The presence of PSHI was low when there were few employees and no labor unions, which is consistent with prior research [7]. This is because small companies and those without unions are thought to be unable to devote resources to safety aspects due to fiscal constraints in a highly competitive environment. As of 2005, the proportion of training investments in all investments by companies with fewer than 30 employees (0.2%) was less than one-eighth that of large enterprises (1.64%) with 1000 or more employees; on that basis, Kang et al. suggested that organizations providing technical support to small-scale workplaces should determine the risks in those workplaces and implement suitable customized safety education [17]. Women and the pink-collar group also had low PSHI status, which may be related to the relatively high proportion of females working in the service sector: In this study, 69.9% of pink-collar workers were women and the occupational injury incidence was highest for the high-risk group in the pink-collar category. This result suggests that PSHI at the workplace is urgently needed, not only for blue-collar workers but also for those in service-type occupations. The PSHI status was low in the group without a need for PPE but, in the group that did need it, a high PSHI status was observed in the workers wearing the PPE, suggesting that PSHI presence may lead to a high PPE usage rate.

With regard to PSHI prevalence, a study based on 2006 KWCS data reported that 4018 out of a total of 6998 workers (57.4%) were listed as having a PSHI [7]. In our 2014 study, there was a slightly higher PSHI prevalence (64.2%). In other words, the absence of PSHI declined from 43.6 to 35.8% over approximately one decade. This may be the result of emphasizing the importance of safety and health education, including the revision of the OSH Act. However, it seems that the absence of PSHI is still high; thus, more rigorous education on the effects of PSHI is needed.

Characteristics associated with occupational injuries differ depending on the injury risk: In the low-risk group, the factors that had statistically significant relationships with occupational injuries were: old age, low education level, low monthly income, blue-collar occupation, long working hours per week, being a temporary worker, physical, biochemical, and ergonomic, as well as the need for PPE. Many results in the low-risk group were consistent with those reported in previous studies. Generally, advanced age and high physical demands at work have been associated with an increased risk of musculoskeletal claims [18], likely leading to occupational injuries. Workers with small incomes and low levels of education had high occupational injury rates. A Korean study that examined different samples during the same year reported that, as workers’ income and educational status increased, their occupational injury experiences decreased [19]. A blue-collar status and long working hours were associated with a high rate of occupational injuries, and construction workers (i.e., blue-collar) in the USA who worked long hours were at high risk of occupational injury [20]. Temporary workers had a lower PSHI status than that of regular workers, and their incidence of occupational injuries was higher, demonstrating the vulnerability of temporary workers to occupational injury [21, 22]. In the present study, physical and biochemical risk factors at the workplace and PPE use were closely related to occupational injuries, which is consistent with results in previous investigations [23, 24]. With regard to PPE use, the proportion of respondents who did not need a PPE was 86.2% in the low-risk group and 47.8% in the high-risk group. PPE status displayed the most significant difference among the study characteristics related to the risk of occupational injuries; therefore, the necessity of wearing a PPE is the most important factor that indirectly indicates the risk of occupational injuries. Generally, shift work is associated with a high occurrence of occupational injury. In a study into the first KWCS dataset, the odds ratio of occupational injuries for workers in shift work was 2.40 (95% CI 1.65–3.50) [25] with similar results reported in overseas investigations [26, 27]. There was a high level of occupational injuries in workers with shift work in the low-risk group, but the result was not statistically significant.

In the high occupational-injury risk group, the factors showing statistically significant associations with occupational injuries were: age, monthly income, working hours per week, tenure, the existence of labor unions, physical, biochemical, PPE usage. Age, monthly income, and working hours per week exhibited no significant relationships with injuries in the high-risk group. Whereas long tenure, absence of labor unions, presence of physical and biochemical risk factors, as well as the need for PPE were associated with an increased occurrence of occupational injuries. There was a high incidence of occupational injuries in workers without labor unions, which can possibly be explained by the relatively few opportunities for individual workers to improve safety and health at work. Notably, PSHI status was significantly low in the absence of unions. Thus, it is necessary to offer options to employees encouraging regular participation in activities to improve safety and health issues at workplaces without labor unions [12]. Being male, in a small company, and ergonomic risk factors were associated with many occupational injuries in a previous study [28], but, in the present study, those relationships were not statistically significant.

Generally, the risk of occupational diseases is higher in smaller companies [29]. This is because workers in small- and medium-sized firms may be exposed to more health-hazard risk factors [30]; it seems that if an organization’s size is large, it can provide safety information more effectively and also systematically control worksite exposure to harmful factors [12]. In previous studies, an increase in injury risk among those who started a new job and an inverse relationship between job tenure and injury risk were observed [31, 32]. However, our study revealed the opposite in the high-risk group with the lowest occupational-injury rate observed in workers with less than 1 year of job tenure. This difference may be due to the particular sampling characteristics or to the control of additional variables in the other investigations. Alternatively, it could be that the more experienced the laborers, the higher the likelihood they work in a more dangerous job. Furthermore, as employees become accustomed to this level of danger, they may be subject to more frequent hazards due to momentary neglect or distraction during work.

In the low-risk group, the relationship between PSHI and occupational injuries was not statistically significant, but this does not indicate irrelevance of PSHI for these workers. We assert that groups differing in their risk of occupational injuries need different approaches depending on their likelihood of falling victim to job-related hazards. The statistically significant differences between the two risk groups were in occupational type, exposure to risk factors, and PPE use. In other words, blue-collar workers with exposure to physical and biochemical risk factors, as well as in need of PPE, are more susceptible to occupational injuries than other employees. The low-risk group results in this study were similar to those in previous studies. However, the high-risk group showed significant associations; mostly for job-related factors including physical, biochemical, and ergonomic risks, and the need for PPE. This indicates that high-risk workers are more likely to be affected by factors that are directly work related, such as the exposure to specific risk factors and wearing a PPE, rather than to more general or indirect factors. Therefore, PSHI might be more important in the high-risk group. This may account for the differences of occurrence of occupational injury between the low-risk group and the high-risk group.

This study has several limitations. First, because the KWCS has a cross-sectional design, the association between PSHI and occupational injuries may be bi-directional and, therefore, causality cannot be established; however, it is very plausible that PSHI has reduced the occurrence of occupational injuries. Second, because this study was based on questionnaires, some limitations, such as a recall bias, may be present in the data. Moreover, a “healthy user bias” could also be present; for example, in the case of critical or fatal injuries, the subject would not be able to respond to the questionnaire. Thus, there is a possibility that the incidence of occupational injuries was underestimated in this study [33]. Nonetheless, there was a statistically significant association between PSHI and occupational injuries in this study, suggesting that PSHI has a greater role in preventing occupational injuries than was expected. Our categorization of a high risk of occupational injury may not have been sufficiently objective; however, considering that it is difficult to judge injury risk as high or low by only assessing the existence and degree of harmful factors, our measurement approach appears rational. Third, we were unable to investigate the details of the occupational injuries such as the nature of the trauma, its severity, treatment, and sequelae.

In spite of these limitations, one of this study’s strengths is its epidemiological nature, which allowed us to examine the relationship between PSHI and occupational injuries in a nationally representative sample of the South Korean population. Fabiano et al. [34] classified the factors influencing occupational accident frequency into (1) technical, (2) economic, (3) labor organizational, (4) environmental, and (5) human, both individual and inter-individual. The KWCS includes these various occupational-injury-affecting factors, and its data were thus appropriate for our investigation. In addition, since the KWCS is conducted every 3 years, follow-up or repeated studies on the associations between occupational injuries and various characteristics might be useful in revealing secular trends and may serve as a basis for future studies into injury reduction in the workplace.

## Conclusions

To prevent occupational injuries, multi-faceted approaches that consider different types and levels of injury risks are needed. Workers with no PSHI and in the high-risk group exhibited an elevated incidence of occupational injuries compared to that in the low-risk group. In the case of occupational-injury in high-risk workers, the provision of more stringent safety education programs is required. Further research is needed to elucidate the factors producing the differences in occupational injuries between the low-risk and high-risk groups, and the influences of PSHI in those groups.

## Abbreviations

CI: Confidence interval
KWCS: Korean working conditions survey
OR: Odds ratio
PPE: Personal protective equipment
PSHI: Provision of safety and health information
